# Can Oxytocin Neuropeptide Promote Human Mesenchymal Stem Cells to Neuron Conversion?: A Novel Approach in Future Neurotherapeutic Research

**DOI:** 10.1021/acschemneuro.5c00520

**Published:** 2025-07-17

**Authors:** Pratikshya Paudel, Rahul Pandit Kumarchinchole, Prabir Kumar Gharai

**Affiliations:** † Department of Chemistry, 7618 Oklahoma State University, Stillwater, Oklahoma 74078, United States; ‡ Department of Animal and Food Sciences, Oklahoma State University, Stillwater, Oklahoma 74078, United States

**Keywords:** Oxytocin, Neuropeptide, Human mesenchymal stem cells, Neuronal trans-differentiation, GSK-3β, Neuroregenerations

## Abstract

Neurodegenerative diseases are characterized by progressive neuronal loss, for which human mesenchymal stem cell (hMSC)-based therapies present a promising regenerative approach. Oxytocin, a neuropeptide known for its neuroprotective and anti-inflammatory properties, has been reported to inhibit GSK-3β in certain models. Given the established role of GSK-3β inhibition in promoting neurogenesis via Wnt/β-catenin signaling, this viewpoint explores the hypothesis that oxytocin could serve as a peptide-based cue to drive neuronal differentiation of MSCs. Consequently, developing effective therapy by emulating the oxytocin sequence could substantially enhance neuroregenerative treatments in neuroregenerative research.

## Graphical abstract

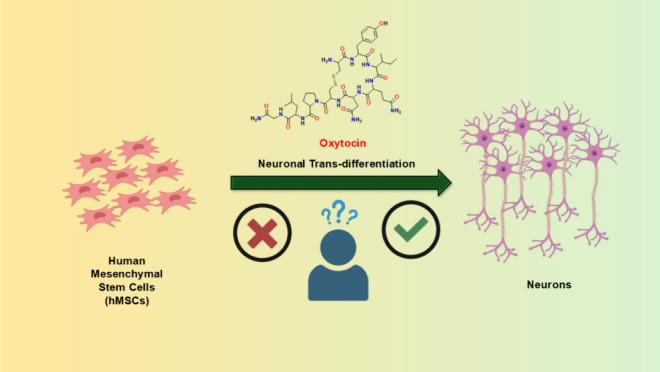

Neurodegeneration refers to the progressive loss of structure and function of neurons in the central nervous system (CNS), which ultimately results in cell death. This decline in neural cells underlies a broad range of neurological diseases, including Alzheimer’s disease (AD), Parkinson’s disease (PD), Huntington’s disease (HD), Stroke, and so on. The clinical symptoms of neurodegenerative diseases are devastating: patients often experience memory loss, impaired cognition, and eventually an inability to carry out basic functions such as speaking or reasoning. At the molecular level, excessive production of reactive oxygen species (ROS), chronic inflammation, and protein aggregates, such as amyloid-beta (Aβ) plaques and hyperphosphorylated tau tangles, are recognized contributors to disease progression.^1^ Among these, tau hyperphosphorylation and Aβ accumulation are particularly well-documented hallmarks of AD. Although extensive research has been dedicated to understanding these mechanisms, there is currently no definitive cure for neurodegenerative diseases. Pharmacological treatments can only provide modest symptom relief, but it does not reverse neuronal loss. As a result, regenerative strategies, especially in stem cell therapy, are gaining interest in repairing nerve damage and restoring function.^2^

Mesenchymal stem cells (MSCs) are multipotent stromal cells capable of differentiating into a range of lineages, including osteoblasts, chondrocytes, adipocytes, as well as neurons. They are useful for therapeutic purposes, because they are easy to collect, unlikely to cause immune reactions, can help regulate the immune system, and have the capacity to migrate to injury sites.^2^ Studies have shown that MSCs can promote antiapoptotic signaling, facilitate axonal repair, and modulate inflammation in CNS injuries.^3^ However, a major limitation to their application in neurodegenerative disease lies in their limited intrinsic capacity to trans-differentiate into fully functional neurons. Currently, trans-differentiation of human MSCs (hMSCs) into neurons often relies on chemical cocktails whose individual components are poorly characterized and may cause unwanted toxicity or adverse effects.^2^ This highlights the need for safer and more targeted approaches to induce hMSCs neurogenesis.

Recent evidence suggests that oxytocin (OXT), a neuropeptide primarily recognized for its role in social bonding, may also influence neurogenesis and neuronal differentiation.^4^ Structurally, OXT is a nonapeptide synthesized primarily in the hypothalamic paraventricular and supraoptic nuclei. It is secreted both centrally and peripherally and exerts its effects through the oxytocin receptor (OXTR), a G-protein coupled receptor expressed in various brain regions, including the hippocampus, amygdala, and cortex.^4^ OXT has traditionally been studied in the context of maternal behaviors, parturition, and lactation, but more recently, its neuroprotective and neurogenic properties have gained attention.

OXT has been reported to exert neuroprotective effects, including reduction of neuroinflammation, oxidative stress, and neuronal apoptosis.^5^ In Alzheimer’s disease models, intranasal administration of OXT has been associated with reduced amyloid-beta accumulation, tau phosphorylation, and cognitive impairment.^5^ Studies have also shown that OXT influences intracellular signaling pathways such as ERK1/2 and GSK-3β.^5^ Separately, inhibition of GSK-3β has been shown to promote neurogenesis through activation of the Wnt/β-catenin signaling pathway.^2^ Although a direct role of OXT in driving neuronal differentiation via GSK-3β inhibition has not been demonstrated, these findings provide a rationale for exploring OXT in the context of stem cell-based neuroregeneration.

GSK-3β, a serine/threonine kinase abundantly expressed in the CNS, plays a critical role in numerous cellular processes, including metabolism, inflammation, and apoptosis.^1^ GSK-3β activity promotes hyperphosphorylation of tau, leading to its detachment from microtubules and the formation of neurofibrillary tangles.^1,2^ Furthermore, GSK-3β is implicated in the upregulation of β-secretase activity, facilitating the production of Aβ from amyloid precursor protein (APP). Inhibition of GSK-3β has been shown to reduce tau phosphorylation, decrease Aβ levels, and enhance adult hippocampal neurogenesis in both *in vitro* and *in vivo* models.^1^ In Wnt/β-catenin signaling pathway, GSK-3β functions as a negative regulator by phosphorylating β-catenin, leading to its degradation. Suppression of GSK-3β activity stabilizes β-catenin, allowing it to translocate to the nucleus and activate transcriptional programs that promote neuronal differentiation.^2^ Consequently, GSK-3β represents a promising molecular target for inducing neuronal differentiation in mesenchymal stem cells. Indeed, small-molecule inhibitors of GSK-3β, such as the imidazole-based compound SG-145C, have been reported to induce hMSC trans-differentiation into neurons by preventing proteasomal degradation of phosphorylated β-catenin.^2^ However, these synthetic inhibitors often require combination with other growth factors and lack long-term safety profiles for clinical use.^2^

Given that OXT has been reported to inhibit GSK-3β activity,^5^ and that GSK-3β inhibition through Wnt/β-catenin signaling is a known route to induce neurogenesis,^2^ it raises the possibility that oxytocin or oxytocin analogs could serve as endogenous, peptide-based inducers of hMSC neuronal-differentiation. While the role of OXT in enhancing neurogenesis and protecting existing neurons is supported by current evidence, there is, to our knowledge, no direct study demonstrating the ability of OXT to transdifferentiate hMSCs into functional neurons. This presents an exciting research gap and a potential future direction for both basic neuroscience and regenerative medicine.

Our current understanding of hMSC trans-differentiation into neurons relies heavily on synthetic molecules and neural growth factors,^2^ whose safety and reproducibility in clinical contexts remain uncertain. The use of oxytocin, a naturally occurring neuropeptide with established safety and documented neuroprotective properties,^4,5^ represents a promising therapeutic alternative. Moreover, the possibility of modifying the oxytocin peptide sequence or designing small-molecule agonists of OXTR with enhanced stability and selectivity could further expand the therapeutic potential of this approach.

From a translational perspective, this concept aligns with the growing interest in single-molecule-based reprogramming strategies, which aim to replace complex, multifactorial induction protocols with biologically relevant cues. By building on the known relationship between OXT, GSK-3β inhibition, and neurogenesis, we propose that oxytocin could serve as a biocompatible and scalable tool for neural reprogramming of hMSCs (Figure 1). If successful, this approach could bridge the gap between *in vitro* stem cell engineering and *in vivo* neuronal regeneration, opening new avenues for treating neurodegenerative diseases.

**Figure 1:**
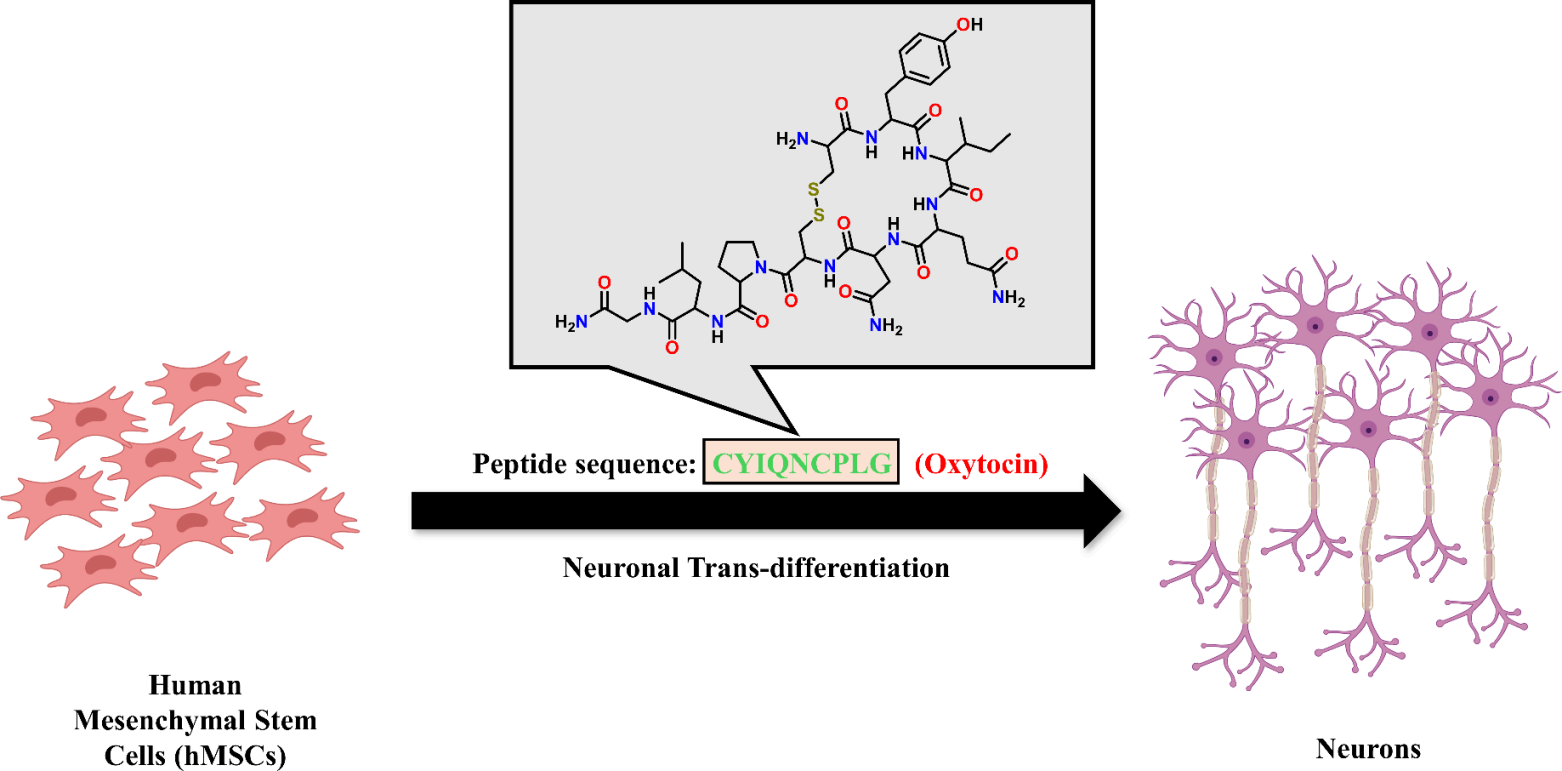
Schematic representation of neuropeptide oxytocin-mediated trans-differentiation of hMSCs into functional neurons.

In conclusion, although the direct role of oxytocin in driving hMSC-to-neuron trans-differentiation has not yet been empirically demonstrated, the converging evidence from studies on OXT’s neurogenic effects, GSK-3β inhibition, and stem cell modulation support this as a testable hypothesis. We suggest that future investigations should focus on validating OXT as a trans-differentiation signal, exploring its downstream signaling in hMSCs, and optimizing delivery methods for potential therapeutic use. Such research could lay the groundwork for a novel class of peptide-based therapies that harness the body’s own signaling molecules to combat neurodegeneration and promote brain repair.
